# The FIELDS Instrument Suite for Solar Probe Plus: Measuring the Coronal Plasma and Magnetic Field, Plasma Waves and Turbulence, and Radio Signatures of Solar Transients

**DOI:** 10.1007/s11214-016-0244-5

**Published:** 2016-03-31

**Authors:** S.D. Bale, K. Goetz, P.R. Harvey, P. Turin, J.W. Bonnell, T. Dudok de Wit, R.E. Ergun, R.J. MacDowall, M. Pulupa, M. Andre, M. Bolton, J.-L. Bougeret, T.A. Bowen, D. Burgess, C.A. Cattell, B.D.G. Chandran, C.C. Chaston, C.H.K. Chen, M.K. Choi, J.E. Connerney, S. Cranmer, M. Diaz-Aguado, W. Donakowski, J.F. Drake, W.M. Farrell, P. Fergeau, J. Fermin, J. Fischer, N. Fox, D. Glaser, M. Goldstein, D. Gordon, E. Hanson, S.E. Harris, L.M. Hayes, J.J. Hinze, J.V. Hollweg, T.S. Horbury, R. A. Howard, V. Hoxie, G. Jannet, M. Karlsson, J.C. Kasper, P.J. Kellogg, M. Kien, J.A. Klimchuk, V.V. Krasnoselskikh, S. Krucker, J.J. Lynch, M. Maksimovic, D.M. Malaspina, S. Marker, P. Martin, J. Martinez-Oliveros, J. McCauley, D.J. McComas, T. McDonald, N. Meyer-Vernet, M. Moncuquet, S.J. Monson, F.S. Mozer, S.D. Murphy, J. Odom, R. Oliverson, J. Olson, E.N. Parker, D. Pankow, T. Phan, E. Quataert, T. Quinn, S.W. Ruplin, C. Salem, D. Seitz, D.A. Sheppard, A. Siy, K. Stevens, D. Summers, A. Szabo, M. Timofeeva, A. Vaivads, M. Velli, A. Yehle, D. Werthimer, J.R. Wygant

**Affiliations:** 1 Space Sciences Laboratory, University of California, Berkeley, CA, USA; 2 Physics Department, University of California, Berkeley, CA, USA; 3 School of Physics and Astronomy, University of Minnesota, Minneapolis, MN, USA; 4 LPC2E, CNRS, 3A avenue de la Recherche Scientifique, Orléans, France; 5 Laboratory for Atmospheric and Space Physics, University of Colorado, Boulder, CO, USA; 6 NASA Goddard Space Flight Center, Greenbelt, MD, USA; 7 Swedish Institute of Space Physics (IRF), Uppsala, Sweden; 8 LESIA, Observatoire de Paris, Meudon, France; 9 Astronomy Unit, Queen Mary, University of London, London, UK; 10 Department of Physics, University of New Hampshire, Durham, NH, USA; 11 Department of Physics, Imperial College, London, UK; 12 Department of Physics, University of Maryland, College Park, MD, USA; 13 Johns Hopkins University Applied Physics Laboratory, Laurel, MD, USA; 14 Space Science Division, Naval Research Laboratory, Washington, DC, USA; 15 University of Michigan, Ann Arbor, MI, USA; 16 Southwest Research Institute, San Antonio, TX, USA; 17 Department of Astronomy and Astrophysics, University of Chicago, Chicago, IL, USA; 18 Astronomy Department, University of California, Berkeley, CA, USA; 19 Praxis Studios, Brooklyn, NY, USA; 20 Earth, Planetary, and Space Sciences, UCLA, Los Angelos, CA, USA

**Keywords:** Coronal heating, Solar Probe Plus

## Abstract

NASA’s Solar Probe Plus (SPP) mission will make the first *in situ* measurements of the solar corona and the birthplace of the solar wind. The FIELDS instrument suite on SPP will make direct measurements of electric and magnetic fields, the properties of *in situ* plasma waves, electron density and temperature profiles, and interplanetary radio emissions, amongst other things. Here, we describe the scientific objectives targeted by the SPP/FIELDS instrument, the instrument design itself, and the instrument concept of operations and planned data products.

## 1 Introduction

The FIELDS instrument on the NASA Solar Probe Plus (SPP) mission is a suite of instruments designed to measure DC and fluctuation magnetic and electric fields, plasma wave spectra and polarization properties, the spacecraft floating potential, and solar radio emissions. FIELDS is one of four selected instrument suites, including the Solar Wind Electron Alpha and Protons investigation (SWEAP) (Kasper et al. 2016), the Integrated Science Investigation of the Sun (IS⊙IS) (Mccomas et al. 2016), and the Wide-field Imager for Solar Probe Plus (WISPR) (Vourlidas et al. 2016). Together, these instruments will measure the plasma, fields, and energetic particle environment of the inner heliosphere to solve the fundamental problem of corona heating and the relationship to the coronal magnetic field. The overall science goals and mission definition are described in Fox et al. (2016). SPP will launch in mid 2018 and begin making science measurements of the inner heliosphere a few months later.

FIELDS will measure the 3-component magnetic field of the corona from ‘DC’ to beyond the electron cyclotron frequency, to fully characterize magnetized plasma waves and to map out the coronal magnetic field and its connection to the Sun. Five voltage probes V1–V5 measure the local plasma potential and can be combined to produce two components of the DC electric field and the fluctuating fields of the third component. Quasi-thermal noise spectroscopy and solar radio emissions to 20 MHz are measured on two components of the fluctuating electric field. The FIELDS instrument draws on heritage from instruments on the Wind, Polar, STEREO (Bougeret et al. 2008), THEMIS (Cully et al. 2008), and Van Allen Probes (Wygant et al. 2013) spacecraft.

In this paper, we describe the FIELDS instrument suite, some aspects of the science goals and measurement requirements, and the instrument operations concept. Since this manuscript was written well before the final instrument was built and delivered, there may be changes to the system before launch.

### 1.1 FIELDS Measurements Requirements

As described in Fox et al. (2016), many of the ideas about coronal heating and solar wind acceleration involve Alfvén waves, collisionless shocks, magnetic reconnection, plasma instabilities, and exospheric physics. *In situ* measurement of these phenomena is critical for the SPP science objectives and places requirements on the performance of the FIELDS instrument. The FIELDS Level 1 measurement requirements flow down from the top level SPP science requirements (Fox et al. 2016) with consideration of the expected plasma conditions over the SPP orbit (described in Sect. 1.2 below). These requirements are summarized shown in Table 1 and drive the overall instrument design, performance, and operations concept.

Further requirements are placed on FIELDS from spacecraft electromagnetic cleanliness specifications, the expected launch environment, and other general environmental issues (thermal, radiation, etc.) as well as overall resources (mass and power).

### 1.2 The Plasma Environment of the Inner Heliosphere

To estimate the fundamental plasma parameters at SPP orbit, we reanalyzed the 11 years of data from the Helios 1 mission; Helios 1 explored the inner Heliosphere from 1 AU down to 0.3 AU, from December, 1974 to January, 1986. The radial evolution of the magnetic field strength, the solar wind speed, the density and temperature, among other parameters were studied and extrapolated to radial distances down to 10 *R_s_*.

**Fig. 1:**
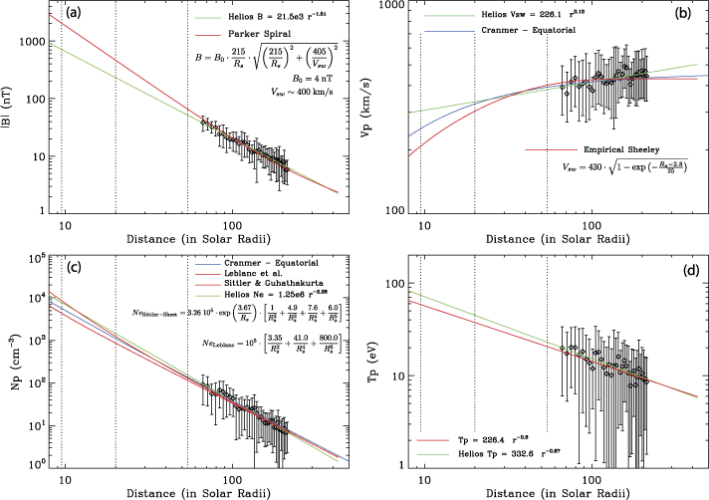
Radial evolution of (**a**) magnetic field intensity, (**b**) solar wind velocity, (**c**) proton density, and (**d**) proton temperature. *Diamonds* represent data from Helios 1 binned in distance, and the *error bars* are the standard deviation for each bin. Model extrapolations are shown in each panel

Figure 1 shows the radial evolution of the magnetic field intensity (Fig. 1a), the solar wind velocity (Fig. 1b), the proton density (Fig. 1c) and the proton temperature (Fig. 1d). The data were binned in distance (bin size of 0.02 AU ≈ 3 · 10^6^ km), and the mean value (diamonds in the plot) and the standard deviation (error bar across each average) computed for each bin. In each panel, the green curve represent a nonlinear least square fit to the Helios data using a simple power law. The vertical dashed lines show the distances of 54, 20 and 10 *R_s_*. The power law for the magnetic field from the Helios data is compared to a Parker spiral field that fits the data between 0.3 and 1 AU (Fig. 1a). The Parker spiral is represented by the red curve: we used an average solar wind speed of 400 km/s and *B*_0_ ≈ 4 nT is obtained from fitting the curve to the data. The power-law for the solar wind speed from the Helios data (green curve in Fig. 1b) is compared the equatorial speed from a model by Cranmer et al. (2007) (blue curve). There are other, empirical models for the radial profile of the solar wind speed (i.e. Sheeley et al. 1997). The red curve represents an empirical optimum *Sheeley-like* model which fits best the Helios data.

The (proton) density is analyzed in a similar way (Fig. 1c). The Helios power-law (green curve) is compared to a few models: the model by Sittler and Guhathakurta (1999) for a density in the sheet (orange curve, which gives the highest estimate at 9.5 *R_s_*), the model by Cranmer et al. (2007) for the equatorial density (blue curve) and the model by Leblanc et al. (1998) (red curve, which gives the lowest estimate at 10 *R_s_*). Compared to the equatorial density, the proton temperature measured by Helios shows a stronger variability. The best power-law (*T_p_* ~ *T*_0_ *r−^α^*) fit to the data (green curve) is a power law with an index *a* ~ 0.67. The red curve is a power-law with an index *a* = 0.6 and a temperature of 9 eV at 1 AU.

Table 2 shows the estimates of the typical slow solar wind parameters at different heliocentric distances over the SPP orbit: at 1 AU (215 *R_s_*), at 55 *R_s_*, and at the final perihelion (~10 *R_s_*). These estimates are based on extrapolation and modeling inward of the Helios results for the magnetic field strength (using the Parker spiral field), and the proton and electron temperature, as discussed above. We used the *Sittler-Guhathakurta* model for the density and the empirical Sheeley-like model for the solar wind speed. At the bottom of the table are estimates of the electric and magnetic fields associated with shocks and current sheets *δB/B*_0_ ~ 1 and *δE/δB* ~ *v_A_* and those of kinetic and dissipative processes at smaller scales (whistlers, Langmuir waves, etc.). These values were then used to estimate spatial scales, frequencies, and convected timescales shown in Table 2, which in turn determine the required performance of the FIELDS instrument.

One of the primary objectives for FIELDS is to measure waves and turbulence in the innermost heliosphere. Of specific interest are very high-time resolution measurements of the fluctuating electromagnetic fields over a wide range of scales, as well as the very large amplitude fields associated with perturbations such as shocks, reconnection magnetic fields, strong whistlers, Langmuir waves, etc.

Some turbulence-driven solar wind models (i.e. Cranmer and van Ballegooijen 2005; Cranmer et al. 2009; Verdini and Velli 2007; Verdini et al. 2010) provide predictions of the radial evolution of solar wind plasma parameters, as well as important fluctuating quantities. The latter are hourly variances of magnetic field and velocity fluctuations. However, the ‘one-hour’ scale does not mean much for the solar wind. Two particular scales that are characteristic of solar wind turbulence are those associated with an observed breakpoint in the turbulent fluctuation power spectrum: (i) the breakpoint between the large, energy injection scales (characterized by a *k*^−1^ or *f*^−1^ power spectrum) and the inertial range scales (characterized by a steeper, *f*^−5/3^ Kolmogorov-like spectrum), and (ii) the breakpoint between the inertial range scales and the kinetic or dissipation scales (characterized with an even steeper, ~ *f*^−3^ spectrum) where energy dissipation is believed to occur.

One way to proceed (which is what we have done here) is to determine how these breakpoint scales or frequencies and their associated turbulence power vary with distance. Helios observations (e.g. Bavassano et al. 1982; Bruno and Carbone 2005) showed that the breakpoint frequency between injection and inertial range scales shifts to higher frequencies closer to the Sun. We re-analyzed Helios power spectra between 0.3 and 1 AU and we found that the breakpoint frequency *f_i_* between the injection and inertial ranges scales as a power-law with distance, *f_i_* (Hz) ~ 4.9 *r*^−1.66^ (*r* in *R_s_*), and so is the turbulence power at those same frequencies, *δB*^2^(nT^2^/Hz) ~ 10^8.1^ *r*^−2^. The scaling of the breakpoint frequency between inertial and dissipation range, *f_d_*, is determined assuming its occurrence for *k ρ_i_* ~ 1 (*ρ_i_* being the proton Larmor radius), with Alfvénic fluctuations propagating with the Alfvén speed *v_A_*, therefore taking into account the proper Doppler shift [2*π f_s/c_* ~ *k* (*v_A_ + V_sw_*)], which leads to *f_d_* ~ *f_ci_* (*v_A_ + V_sw_*)/*v_thi_*.

**Fig. 2:**
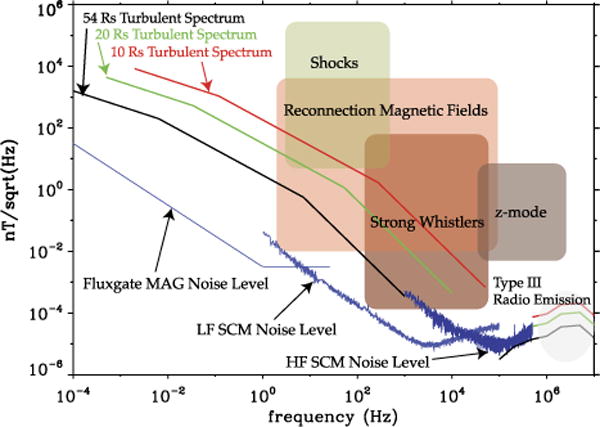
Estimates of the magnetic field fluctuation spectra at 54, 20 and 10 solar radii, together with the instruments MAG and SCM noise levels. These expected turbulence levels were obtained from breakpoint scalings. Also shown are the expected magnetic field fluctuation levels from shocks, reconnection fields, strong whistlers and z-modes over the SPP orbit

Combining these findings and assuming the above-mentioned scaling for the magnetic field power spectra at injection, inertial and dissipation scales, we can determine the expected magnetic field spectra at 54, 20 and 9.5 *R_s_*. This is illustrated in Fig. 2. Also shown on this plot are the expected SPP fluxgate and search coil magnetometers noise floor. The predictions of magnetic field levels associated with shocks, reconnection, strong whistlers and z-modes (with Langmuir wave amplitude) are scaled from 1 AU observations based on the scaling of fundamental plasma parameters (inertial length, field strength, etc.) with radial distance. The type III radio emission estimate assumes free propagation (*B = E/c*).

### 1.3 Spacecraft Charging and Plasma Wake Formation

An issue identified early in the instrument definition phase is that of spacecraft charging and plasma wake formation. It is well known that a sunlit spacecraft will charge positively and that the charge cloud will act as an electrostatic obstacle to incoming ions (especially when *T_e_* ≳ *T_i_* as in the solar wind), creating a plasma wake in the anti-flow direction.

Over the SPP orbit, photoemission currents from sunlit surfaces exceed by factors of 10–100 the electron currents from the plasma. Typically, sunlit surfaces charge positively and the lowest energy photoelectrons are attracted back to it. The charging continues until the fluxes of photoelectrons with energies greater than this potential (and therefore, able to escape to infinity) are equal to the fluxes of incoming plasma electrons. In equilibrium, surface potentials are typically 5 to 10 volts.

Surfaces that are not in sunlight are expected to charge negatively. In the absence of photoelectron currents, the incoming electron fluxes exceed the incoming ion fluxes. In equilibrium, the potential of the shadowed surface becomes negative at a few times the electron temperature, which may be several hundred volts on SPP. To prevent this situation from arising on SPP, the sunlit portions of the spacecraft are electrically connected to the shadowed surfaces so that the entire spacecraft surface is near electric equipotential. This electrical conductivity is guaranteed by the imposition of an electrostatic cleanliness specification; the photo-emitting surface on SPP is the heat shield, so it must be conductive and electrically tied to the spacecraft body. For SPP and most spacecraft, the photo-emitting surface area is a reasonable fraction of the total spacecraft surface area, so photoemission can dominate plasma electron collection by the entire spacecraft such that the equilibrium potential of the spacecraft will not be dramatically negative.

The electric field sensors on SPP are expected charge to a positive potential with respect to the nearby plasma unless a bias current is applied to bring their potential close to that of the nearby plasma. It is advantageous to apply such a bias current because the Langmuir probe curve of a sensor has its minimum *dV/dI* (*V* is electric potential and *I* is current) at the plasma potential, which means that current imbalances between opposite sensors (the main source of error in the measurement) produce the minimum error in the electric field measurement.

The above discussion is oversimplified as it neglects effects such as secondary electron emission, wakes, etc. (Whipple 1981). It is also oversimplified because it neglects geometric effects that may be important in the SPP mission, as next discussed. Near perihelion, the photoelectron density near the surface of the flat heat shield is large because of the intense sunlight, so the photoelectron Debye length is small compared to the dimensions of the heat shield. These conditions can create an electrostatic barrier near the heat shield through which newly created photoelectrons do not have sufficient energy to escape but incoming plasma electrons, being much more energetic, can penetrate to the surface of the heat shield. As a result the heat shield and the spacecraft may charge to large negative voltages even if the electrostatic cleanliness specification is satisfied (Ergun et al. 2010; Guillemant et al. 2012; Donegan et al. 2014). Under these conditions the bias current to the electric field sensors will need to change. A flight software algorithm is implemented to make such changes.

Even with electrostatic cleanliness and bias current control, further errors in the measurement may occur due to wake effects. At almost all regions and times along the SPP orbit, the solar wind speed relative to the spacecraft exceeds the ion thermal speed, creating a high Mach flow relative to ions. On the other hand, electron thermal speeds almost always exceed the solar wind speed. As a result, an ion cavity is formed on the downstream side of the spacecraft, which is partially filled by the electrons creating a negative potential in the spacecraft wake. The potential in the wake (*V_wake_*) can be a significant fraction of the electron temperature (*k_B_T_e_*) or higher.

**Fig. 3:**
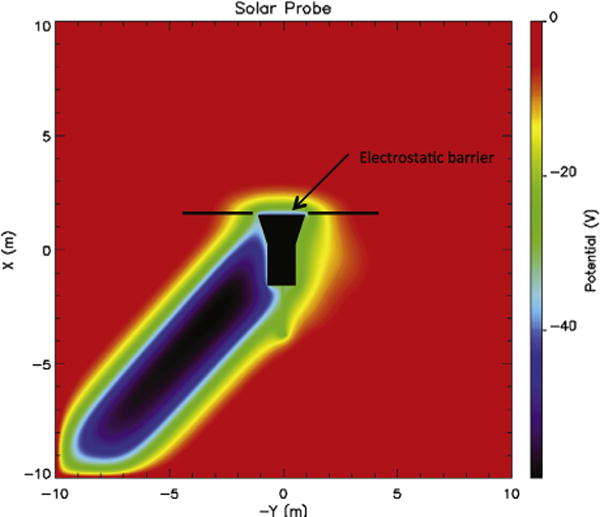
The electric potential surrounding a simple model of the SPP spacecraft near perihelion [see Ergun et al. 2010 for details on the simulation]. The X-direction is toward the sun. The Y-direction is the orbital track of the spacecraft. *Color* represents potential in volts. The *thick black lines* depict the electric field antennas. The plasma density is 7000 cm^−3^, the electron temperature is 85 eV and the ion temperature is 85 eV. The solar wind speed is 200 km/s and the spacecraft orbital speed is 180 km/s. The plot is in the frame of the spacecraft

An example of the spacecraft wake is displayed in Fig. 3. This figure shows a slice of the expected wake in the SPP orbital plane. The image is in the frame of the spacecraft under representative conditions at perihelion (roughly 10 *R_S_*). The sun is at the top (X-direction) and the spacecraft orbit is along the Y-direction. The spacecraft velocity of 180 km/s is nearly the same as that of the solar wind, so the wake is at rough 45° from the sun. The wake has a minimum potential of ~−60 V, about 70 % of *k_B_T_e_*.

The ion wake has two unwanted effects on the electric field measurement. It creates an electric field in the *X–Y* plane, which can and should be detected by the electric field instrument. Near the wake, the unwanted wake electric field can exceed that of the solar wind making it difficult to isolate the naturally occurring electric field. Wake formation provided the primary motivation to locate the FIELDS electric field antennas as far forward (toward the sun) on the SPP spacecraft as possible to minimize its influence. The forward location of the antennas greatly reduces the wake-induced electric field.

The wake also can alter the spacecraft potential. It contacts nearly one half of the spacecraft surface. The negative potential of the wake prevents nearly all secondary electron escape (secondary electrons have 2 eV characteristic energies) from the downstream facing surfaces of the spacecraft but allows a significant part of the thermal electron current to reach the spacecraft (*T_e_* is expected to be 50–100 eV at 10 *R_S_*). This effect deepens a possible negative charging of the SPP spacecraft.

Figure 3 also shows the electrostatic barrier formation on the shield (thin blue region on the +X side of the spacecraft). This barrier blocks a surprisingly large fraction (nearly 90 %) of the photoelectron fluxes from escaping the sunward side of the spacecraft, which is the primary cause of the spacecraft charging negatively (−25 V). This barrier has a beneficial influence on the electric field measurement as it creates a symmetric potential structure about the spacecraft near the electric field antennas. However, it may have a detrimental effect on measurements of the electron distributions. Since the electrostatic barrier results from a nonlinear and non-monotonic potential structure, the depth and influence of the barrier are difficult to quantify even with simulation.

## 2 Instrument Design

The FIELDS instrument combines magnetic and electric field measurements into a single, coordinated experiment. Magnetic fields are measured using both fluxgate and search-coil (induction) magnetometers mounted on a deployable boom in the spacecraft umbra. FIELDS will make electric field measurements both as a current-biased resistively-coupled doubleprobe instrument (Harvey et al. 1995; Bonnell et al. 2009; Wygant et al. 2013) *and* as a capacitively-coupled radio and plasma wave instrument (Bougeret et al. 1995, 2008). This places several constraints on the geometry and surfaces of the antenna system, and upon the design of the preamplifier and receiver electronics. In addition to the primary measurement objectives of electric and magnetic fields and waves, the FIELDS measurements will provide very accurate electron density and temperature measurements, density and velocity fluctuation measurements, and signatures of dust impacts on the SPP spacecraft.

**Fig. 4:**
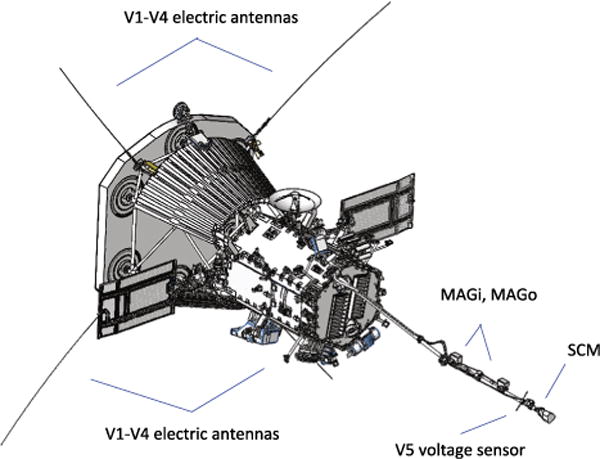
FIELDS uses 5 voltage and 3 magnetic sensors to measure electric and magnetic fields. The four V1–V4 sensors are deployed into full sunlight near the base of the SPP heat shield (TPS). A search coil magnetometer (SCM) is mounted at the end of the instrument boom. Two fluxgate magnetometers (MAGi and MAGo) and a simple voltage sensor V5 are also mounted on the boom

Figure 4 shows the general layout of the FIELDS sensors on the spacecraft. The V1–V4 electric field probes are mounted at the base of the SPP Thermal Protection System (TPS) or heat shield and deploy out into full sunlight. At the SPP perihelion altitude of 9.8 *R_S_* these antennas will reach temperatures of more than 1300 °C. Another simple voltage sensor V5 is mounted on the magnetometer boom in the umbra of the spacecraft. Two fluxgate magnetometers (MAGi and MAGo) and a search coil magnetometer (SCM) are also mounted on the boom. These sensors are described further below.

**Fig. 5:**
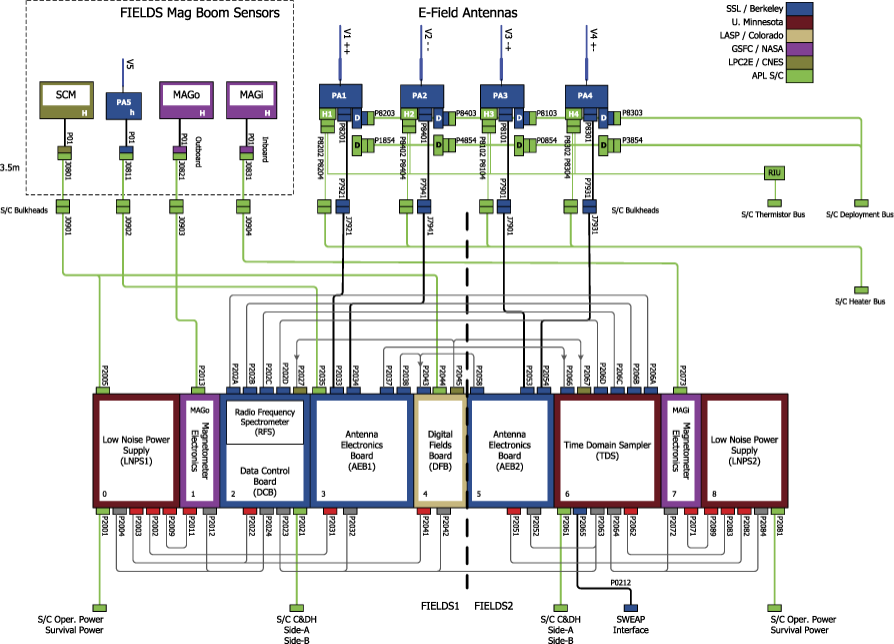
The FIELDS suite block diagram. Boom-mounted sensors are diagrammed in the *upper left dotted box*; these sensors are fixed to the boom and deploy with it. The four TPS-plane electric field/voltage sensors are deployed by actuation from the spacecraft. The Main Electronics Package (MEP) at the bottom is mounted within the spacecraft body and consists of two sides—FIELDS_1 and FIELDS_2—providing some redundancy in the case of power supply or computer failure. FIELDS also has a dedicated interface to the SWEAP instrument. Color-coding indicates institutional responsibility of each hardware component

A block diagram of FIELDS is shown in Fig. 5. In addition to the sensors listed above, FIELDS consists of three digital signal processing boards, a computer/processor unit, two boards to control antenna biasing, magnetometer formatting electronics, and two low noise power supply units. FIELDS was originally proposed with single string architecture for the processing computer and power supply. A failure mode analysis of measurement requirements performed in Phase B, showed that a single failure in FIELDS could lead to the loss of an unacceptable number of mission-level science requirements. As a result, the FIELDS instrument architecture was split into two halves such that no single failure results in a loss of all measurements. The subsystems and the two-sided architecture are described further below.

The FIELDS instrument is very sensitive to conducted and radiated noise sources from other instruments and spacecraft subsystems; at high frequencies (~MHz) the instrument is sensitive at the level of $nV/\sqrt{Hz}$ (cf. Sect. 2.2.4). To accommodate this, a spacecraft level electromagnetic cleanliness (EMC) program has been established. A fundamental requirement of the SPP EMC program is that all DC–DC power converters be operated at fixed frequencies in 50 kHz intervals, beginning at 150 kHz (i.e. 150 kHz, 200 kHz, 250 kHz, etc.) and that these chopping frequencies be crystal-controlled. This picket-fence approach concentrates power-supply noise and harmonics into well known and narrow frequency bands providing ‘clean’ regions of spectral density in which to make sensitive measurements (see Sect. 2.2.4 and Fig. 16). Further, the FIELDS instrument team made the decision to synchronize its internal sampling clocks to multiples of 150,000 Hz with its master clock operating at 150,000 Hz × 256 = 38.4 MHz. To accommodate digital signal processing algorithms, which prefer power-of-two data blocks, FIELDS uses a rescaled timebase that we call a ‘New York second’ (NY sec) and define 1 NYsec as a convenient power-of-two number of clock cycles (2^17^) of the standard 150 kHz power supply chopping frequency. Thus 1 NYsec is defined as being 2^17^/150,000 ≈ 0.873813 … sec. While the FIELDS DFB ‘burst mode’ operates in sync at 150,000 Sa/s (samples/second), which is 2^17^ samples/NYsec, the lower cadence data is sampled at rates that are 150,000 Sa/s divided by further powers-of-two. For example, the fluxgate magnetometers operate in synchronization at 150,000/2^9^ ≈ 292.969 Sa/s, which is exactly 256 Sa/NYsec. This allows FIELDS to sample in synchronization with the EMC prescribed frequency of 150 kHz avoiding large noise signals from power converters *and* to maintain the power-of-two data format desired by Fast Fourier Transform (FFT) algorithms. Note that the FIELDS and SWEAP (Kasper et al. 2016) instruments use a master/slave clock configuration over a dedicated interface: SWEAP uses the FIELDS clock signal when available. This will maintain phase coherence between the FIELDS and SWEAP measurements, enabling both accurate high cadence data processing *and* the removal of deterministic noise signals.

### 2.1 Sensor and Preamp Design

The FIELDS suite uses five voltage probes and three magnetometers to make measurements over 20 MHz of bandwidth and 140 dB of dynamic range. The ‘V1–V4’ voltage probes described in Sect. 2.1.1 function both as current-biased double probe electric fields sensors (as on the THEMIS or Van Allen Probes satellites) *and* as capacitively-coupled radio and plasma wave sensors (as on Wind, Cassini, STEREO). The V5 sensor described in Sect. 2.1.2 makes a simple voltage measurement near the magnetometer boom and may be used to infer the sunward electric field for plasma waves. The fluxgate and search coil magnetometers are standard devices for measuring low frequency and wave magnetic fields. These sensors are describe in the sections below.

#### 2.1.1 The V1–V4 Electric Antennas

Four voltage sensors (V1–V4) are deployed in nearly orthogonal, co-linear pairs slightly behind the plane of the spacecraft heat shield (the Thermal Protection System or ‘TPS’), as shown in Fig. 4. To function properly as a double-probe electric field measurement, these sensors must be coupled to the plasma through a photoelectron current; i.e. they must be in sunlight. This orientation also keeps the V1–V4 antennas out of the spacecraft wake and ensures a minimal perturbation to the spacecraft interaction with the solar wind.

**Fig. 6:**
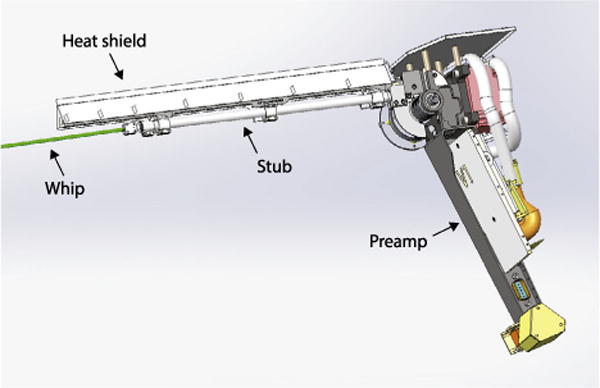
A CAD drawing of a V1–V4 antenna unit. The whip (colored *green* here) is the sensor and extends 2 meters beyond the end of the 30 cm stub. The stub acts as an electrical and thermal isolator. The niobium C103 whip signal is carried back through a small, pure niobium wire contained in the stub to the preamplifier at the base. A heat shield shadows the stub, allowing it to radiate excess heat from the whip, while another shield supports blanketing that blocks heat radiating from the TPS

Figure 6 shows a CAD drawing of one of the units; each unit is identical, with some small differences in the mounting hardware. The primary sensor consists of a 2 m long, 1/8″ diameter Niobium C-103 thin-walled tube (called the ‘whip’). All but the last 8 cm of the whip are exposed to full sunlight and will reach high temperatures (> 1300 °C) at SPP perihelion. The whip is clamped to a 30 cm Molybdenum ‘stub’ element which acts as an electrical and thermal isolator and the signal from the whip is fed through with a molybdenum wire to the hinge and preamp below. A chevron shaped C-103 heat shield covers the final 8 cm of the whip and the entire stub. This creates a substantial shadowed area that radiates excess heat into space. Additionally, the shield, whip, and stub are all isolated from each other with sapphire, a good thermal insulator. Both of these features greatly reduce the thermal input into the base, where conductors and other materials must be below 230 °C. This design has been modeled and verified in the laboratory. The preamp sits near the bottom of the antenna mechanism, to minimize stray capacitance from the cables. The antenna system is stowed back against the spacecraft for launch and deploys by spring force and is rate-limited by a flyweight brake system. After release, it will take a few seconds to deploy to the final configuration. Each sensor will be deployed individually, with the FIELDS electronics operating, to aide in calibration and characterization of the measurement.

#### 2.1.2 The V5 Voltage Sensor

A simple voltage probe (‘V5’) will be mounted on the SPP magnetometer boom, deployed in the umbra behind the spacecraft (and is therefore coupled to the plasma through thermal electrons rather than photoelectrons). While this sensor sits in the spacecraft plasma wake (described above) and will see the low frequency signatures of that interaction, it will provide good capacitively-coupled voltage measurements of the radial electric field *E*_‖_ present in plasma waves and will help constrain the knowledge of the electrostatic center of the spacecraft.

**Fig. 7:**
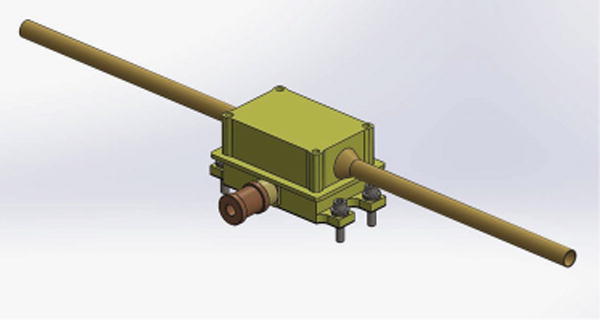
A CAD drawing of the V5 voltage sensor. Two short tubes act as a single (electrically tied) sensor to measure the plasma voltage. A simple preamplifier is housed in an attached enclosure. The tubes can be current-biased and the preamp enclosure can be voltage-biased

The V5 design is shown in Fig. 7. Two short sensor elements extend from a preamplifier box and are electrically isolated from the box by PEEK fittings. The two tube elements are tied together at the preamp (i.e. it is not a differential measurement).

#### 2.1.3 Electric Preamplifiers

**Fig. 8:**
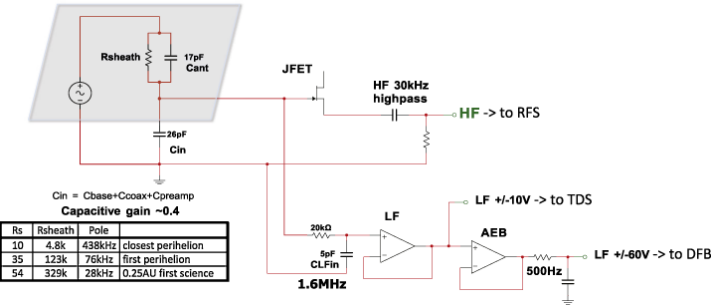
A simplified schematic of the V1–V4 electric preamplifier circuit. A signal from the antenna whip is fed into 3 separate channels that feed the DFB, TDS, and RFS receivers. The LF side using a floating voltage system to accommodate the large expected plasma voltage variations. The *grey box in the upper left* represents the plasma voltage signal and sheath impedance, and some estimated values of the sheath resistance are shown in the table within the figure

The four main antennas are connected individually to the V1–4 preamps and the fifth antenna on the mag boom is connected to the V5 preamp. The preamps provide low-noise high-impedance inputs, voltage gain, and low impedance outputs. As shown in Fig. 8, the V1-4 preamps provide three outputs: the HF 20 MHz bandwidth output to the Radio Frequency Spectrometer (RFS), the MF 1 MHZ output to the Time Domain Sampler (TDS), and the LF 64 kHz output to the Antenna Electronics Board (AEB) and Digital Fields Board (DFB). The HF amplifier chain is a new design consisting of a FET input buffer followed by a wide bandwidth op amp providing gain and driving a 50 ohm terminated coax output. The LF and MF signals are provided by a second unity gain op amp, as used in legacy designs (e.g. THEMIS, RBSP). This op amp is powered by a supply referenced to a “floating ground driver” on the AEB (see Sect. 2.2.1 below), and provides a signal range of ±70 V from DC to 300 Hz and ± 10 V from 300 Hz to 1 MHz. The V5 preamp does not include the HF chain and consists of the single legacy op amp design providing LF and MF outputs.

#### 2.1.4 Fluxgate Magnetometers

The two fluxgate magnetometers (MAGs) for SPP are similar to the triaxial, wide-range, low-power, and low-noise magnetometers built by Goddard Space Flight Center (GSFC) for MAVEN, Van Allen Probes, STEREO, etc. This line of flight magnetometers totals 79 instruments to date starting with IMP-4, launched in 1966. For SPP, the MAGs will provide data with bandwidth of ~ 140 Hz, sampling at 292.97 Sa/s, as part of the FIELDS instrument suite. The measurement dynamic range is ±65,536 nT with a resolution of 16 bits. The primary science objectives addressed by the magnetometers are determining the structure and dynamics of the magnetic fields at the sources of the fast and slow solar wind, contributing to the study of the coronal processes that lead to heating of the solar corona, and exploring the roles of shocks, reconnection, and turbulence in accelerating energetic particles.

**Fig. 9:**
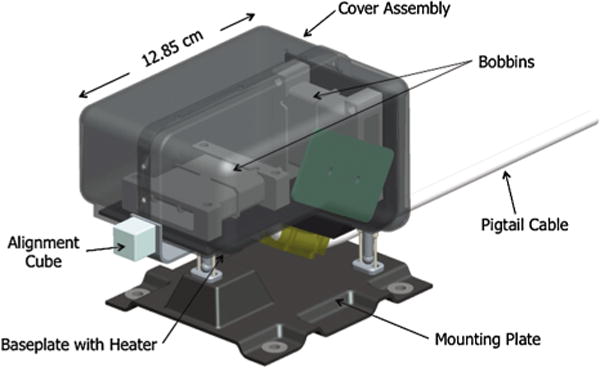
CAD drawing of a SPP MAG sensor, showing the composite structure supporting the two bobbins and electronics board (*green*) inside the composite cover. Also seen are two of the three kinetic mounts supporting the sensor on the composite 4-hole mounting plate, the alignment cube, and the pigtail harness that connects to the spacecraft harness

The sensor design for the SPP MAGs (Fig. 9) will provide maximum thermal isolation from the boom, which will undergo considerable temperature variation, depending on its location in the umbra or in sunlight. Kinematic mounts limit the heat transfer across the feet of the sensor. The heritage of the kinematic mounts derives from the Juno magnetometers, where they were used to ensure that the sensor temperature variation was limited. Heater power is provided by a proportional AC heater to reduce the temperature variations of the sensors and to provide survival heating. The heater is synchronized to a frequency provided by the MEP. The sensor mass requirements have lead to use of a lightweight composite base.

Each MAG sensor has a corresponding electronics board in the SPP FIELDS MEP. For enhanced reliability, the outer MAG board is controlled by the DCB, and the inner MAG board by the TDS. The control component on each board is the rad-hard Aeroflex FPGA that contains all of the MAG logic functions and on-board SRAM. These functions include command handling, telemetry packet formatting, ADC readout, auto-ranging algorithm, DRIVE clock generation, and housekeeping readout. MAG produces a data product at the required cadence of 1 message per 0.874 seconds. The ranging algorithm selects one of the four ranges (±1024, ±4096, ±16,384, ±65,536 nT) based on the ambient magnetic field. The sensor connection to the electronics boards is accomplished by a tuned circuit, including the harness, which is calibrated at the GSFC Acuña Magnetometer Test Site. Because the sensor temperatures will be substantially lower than standard GSFC magnetometer sensors and will vary significantly due to changes in the spacecraft Mag boom orientation relative to the Sun, calibration will be performed over a wide range of temperatures.

**Fig. 10:**
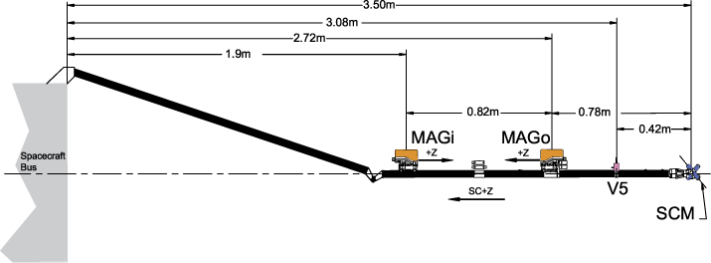
A schematic of the spacecraft magnetometer boom and sensors, shown deployed. Two fluxgate magnetometers are located at 1.9 m (MAGi) and 2.72 m (MAGo) from the rear deck of the spacecraft. The V5 voltage sensor is at 3.08 m and the search coil magnetometer (SCM) is located at the end of the boom: 3.5 m from the spacecraft. This is a relatively short boom, constrained to remain in the spacecraft umbra at perihelion. SCM data will require special processing to remove the drive signal from the fluxgates

The MAG sensors are mounted on the SPP magnetometer boom (Fig. 10), where their relative separation and close proximity to the spacecraft compromise their gradiometric functionality, and thus the ability for an accurate removal of any spacecraft field contamination at the outer MAG. This enhances the importance of the magnetic cleanliness testing being implemented for the spacecraft and payload.

#### 2.1.5 Search Coil Magnetometer

The search coil magnetometer (SCM) will measure all three components of the AC magnetic signature of solar wind fluctuations, from 10 Hz up to 50 kHz and a single component from 1 kHz to 1 MHz. The wide bandwidth and dynamic range allows FIELDS to investigate transients caused by interplanetary shocks and reconnection, the turbulent cascade beyond the electron kinetic scale, but also numerous plasma wave modes.

**Fig. 11:**
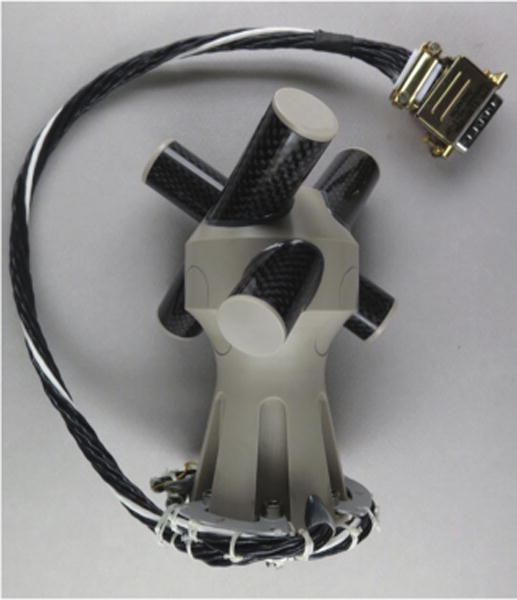
Engineering model of the search-coil magnetometer (SCM) for SPP

The SCM instrument consists of a triaxial search-coil that has a solid technical heritage in several past missions (Dudok de Wit et al. 2011). Nearly identical instruments are being built for the Taranis and Solar Orbiter missions. Each sensor consists of a magnetic core with a winding whose voltage is proportional to the time-derivative of the magnetic field (Seran and Fergeau 2005). Two sensors of SCM cover the ELF/VLF frequency range from 10 Hz–50 kHz. The third one is a dual-band sensor that covers both the ELF/VLF and the LF/MF (1 kHz–1 MHz) ranges. The three sensors, each of which is 104 mm long, are mounted orthogonally on a non-magnetic support, see Fig. 11.

Two challenges raised by Solar Probe Plus are the low temperature environment on the magnetometer boom, and the unusually large dynamic range of the instrument. The latter is needed to accommodate both small-amplitude fluctuations of solar wind turbulence, and large transients near the Sun. Peak values, as scaled from observations made by Helios at distances from the Sun down to 0.29 AU, may reach 3000 nT in the ELF/VLF range. Thanks to a careful design, the dynamic range of the instrument has been increased from past models by several tens of dB, now reaching 160 dB in the ELF/VLF range, and 130 dB in the LF/MF range. SCM will be located in the shade of the spacecraft, at the end of the magnetometer boom, and thus needs a heater to keep it above deep space temperatures. To mitigate thermal losses, the instrument will be wrapped in an insulating MLI layer, with very compact design. The SCM design is very compact; in particular, the preamplifier has been miniaturized by 3D Plus, and will be located inside the foot of the instrument.

**Fig. 12:**
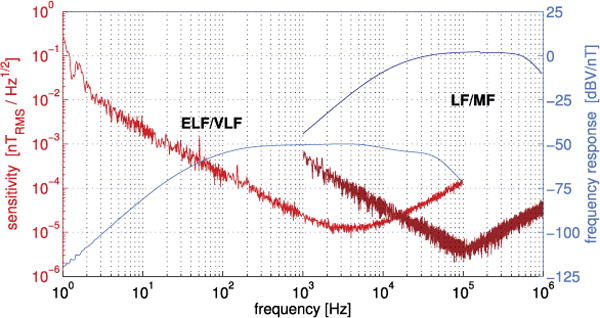
Measured sensitivity (*in red*) and frequency response (*in blue*) of SCM. The *curves on the left* are for the ELV/VLF antenna and the *curves on the right* for the LF/MF one. The highest measurable levels are 3000 nT in the ELF/VLF range, and 100 nT in the LF/MF range

The sensitivity and instrument response of the SCM are illustrated in Fig. 12. The sensitivity is sufficient to observe small-amplitude solar wind turbulence in the inner heliosphere, and properly distinguish Elsässer variables, while also capturing large transients (hence the low gain −50 dBV/nT in the ELF/VLF range). The analog signals in the ELF/VLF range will be processed by the Digital Fields Board (DFB), which will deliver either spectra or continuous waveforms up to 150,000 Sa/s. The LF/MF signal will be processed by any of the RFS, DFB, or TDS receivers. The survey data products will be spectral matrices, which give access to the polarization, and waveforms of up to 293 Sas/s for all three components. The latter will be merged with the DC magnetic field, as measured by MAG, into one single composite magnetic field product.

### 2.2 Main Electronics Package

**Fig. 13:**
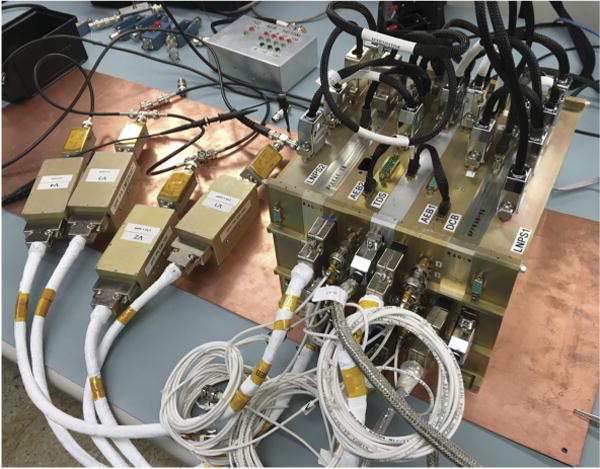
A photograph of the engineering model (EM) of the FIELDS main electronics package and V1–V4 preamps. The individual boards are labeled

The Main Electronics Package (MEP) is the stack of FIELDS receivers, computers, and power supplies and is mounted within the SPP spacecraft structure. Figure 13 shows the engineering model (EM) MEP on the bench in Berkeley. The electronic boards in the MEP are described below.

#### 2.2.1 Antenna Electronics Board (AEB)

The signals from the four V1–V4 electric field antennas and the V5 sensor enter the MEP on the Antenna Electronics Boards (AEB1 and AEB2). At this point, the DC signal has a gain of near unity and a dynamic range of 115 volts. The sensor electric field signals are transferred to the Digital Filter Board (DFB), the Time Domain Sampler (TDS) and the Radio Frequency Spectrometer (RFS) for signal processing and digitization. These two AEB units are functionally almost identical with AEB1 on the FIELDS1 side of the instrument and AEB2 on FIELDS2 (see block diagram above). AEB1 processes signals from V1, V2, and V5, while AEB2 processes V3 and V4 signals.

One of the principle functions of the AEBs is to generate and transfer the different control voltages that are used for current biasing of the probe and to control the potential of surfaces near the sensors. These signals are transferred to each of the five sensor units. On the forward sensors (V1–V4), these bias control circuits consist of the whip current bias circuit, and the stub and shield voltage bias circuits. On the tail sensor (V5), these bias control circuits consist of the antenna bias current circuit and the stub (box) voltage bias circuit.

The current bias circuitry implements the injection of a microprocessor-controlled bias current from the sensor surface into the plasma to control the sensor floating potential and the plasma sensor-sheath resistance. This is achieved by setting the operational point on the current-voltage curve of the sensor/plasma sheath. In low-density plasmas, the bias current is generally adjusted to be a significant fraction of the total photocurrent to the probe. The broad range of illumination levels achieved on SPP, ranging from 16 to over 500 times the solar constant at 1 AU, require the implementation of three ranges for the bias current on the illuminated sensor surfaces (V1–V4). These three ranges are controlled to 12-bit accuracy (0.025 % of full range) as follows: ±802 nA, ±14.1 μA, ±414 μA.

The current bias circuit has a bandwidth (3-dB) of 450 Hz. The current bias circuit on V5 only implements the lowest range (±802 nA). The voltage bias circuits (stub and shield on V1–V4; box on V5) allow for ±40 V of DC offset relative to the sensor potential to be applied to the given surface for sensor-to-spacecraft potential differences of ±60 V DC, primarily for photo- and secondary electron control. The voltage bias circuits have the same bandwidth (3-dB) as the current bias circuits (450 Hz).

The optimum bias currents and voltages will be determined by on-orbit bias sweeps during both the commissioning phase near 1 AU (for comparison with prior mission results) and also at a lesser cadence throughout the perihelion passes. It is expected that the bias currents and voltages will be changed using explicit commands during the inbound and outbound legs of the perihelion passes. In addition, an on-board automated bias current adjustment system successfully used in flight on the NASA Van Allen Probes mission (Wygant et al. 2013) may be used if perihelion conditions prove to be too dynamic for fixed bias settings to encompass.

The commanded values (digital to analog converter settings) controlling the bias currents of the whips and the bias voltages of the stubs and shields are included in instrument housekeeping telemetry as are the parameters of the bias sweeps. In addition to generating and controlling the sensor bias currents and voltages, the AEB also houses the floating ground driver and floating power supplies used to power the sensor preamplifiers (LF/MF stage and HF front-end), as well as additional regulation for the HF backend. This floating preamp design allows for the use of low-voltage, low-noise, low-leakage parts in the preamp design while still allowing the system to handle the tens of volt quasi-DC offsets between sensor and SC potentials due to current biasing and differences in sensor and spacecraft illumination. The floating ground driver has a dynamic range of ±100 V (required range is ±60 V), with a bandwidth (3-dB) of 450 Hz.

#### 2.2.2 Digital Fields Board (DFB)

The Digital Fields Board (DFB) is responsible for conditioning, digitization, and processing signals from the five voltage sensors and four search coil magnetometer (SCM) coils over a frequency range of DC to ≈60 kHz, as well as production of a calibration signal to the SCM. These nine analog inputs are processed by the DFB into twenty-five digital data streams, which are then used to produce a range of time- and spectral-domain data products.

The signals measured by the FIELDS five voltage sensors are amplified by the preamplifiers and are passed to the AEB boards for low frequency signal conditioning before entering the DFB. Four analog voltage signals arrive directly from the SCM sensor, three from the low frequency (LF) SCM windings, and one from the medium frequency (MF) winding. These nine inputs are processed by the DFB into twenty-six digital data streams, which are then used to produce a wide range of time-domain and spectral-domain data products. A more detailed description of the DFB for Solar Probe Plus can be found in Malaspina et al. (2016).

**Fig. 14:**
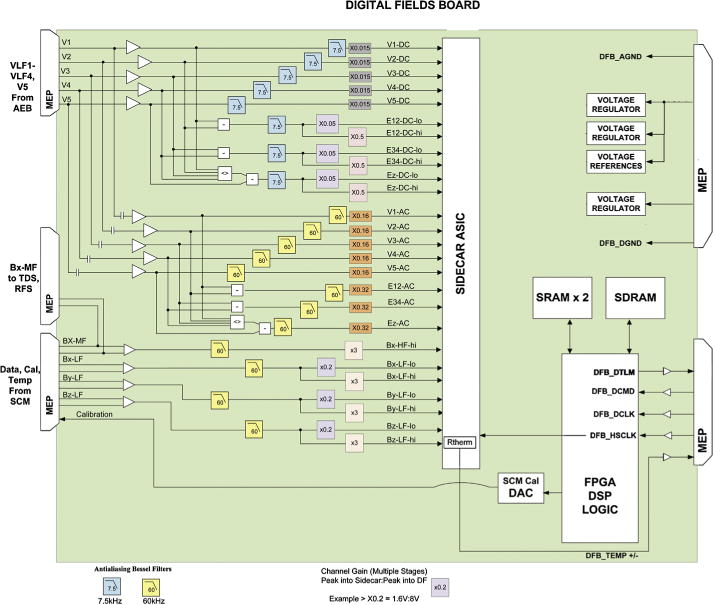
A block diagram of the DFB. The DFB processes 26 input signals into a Teledyne SIDECAR ASIC at 150 kSa/s and performs digital signal processing to produce spectral and cross-spectral matrices, in addition to time series data

Voltages measured by the five FIELDS antennas encounter the FIELDS preamplifiers and the AEB boards before entering the DFB. The four SCM signals arrive directly from the SCM sensor, three from the low frequency (LF) SCM coils, and one from the medium frequency (MF) coil. DFB analog conditioning includes separation into DC-coupled and AC-coupled signals, production of differential signals, application of anti-aliasing filters, and application of gain stages. Figure 14 summarizes these steps graphically.

The five antenna signals are divided into DC-coupled and AC-coupled using a singlepole high-pass filter (−3 dB at 100 Hz). Both DC-coupled and AC-coupled differential signals are produced using voltages measured by the antennas in the plane of the heat shield: *E*_12_ = *V*_1_ − *V*_2_ and *E*_34_ = V_3_ − *V*_4_*.* DC- and AC-coupled differential signals along the axis of the spacecraft are also generated: *E_z_ = V*_5_ − (*V*_1_ *+ V*_2_ *+ V*_3_ *+ V*_4_)/4*.* Four-pole low-pass Bessel anti-aliasing filters are then applied to all *V* and *E* signals (−3 dB at 7.5 kHz for DC, −3 dB at 60 kHz for AC). DC-coupled E signals are separated into three low gain and three high gain channels, with a 10 × relative gain difference. Six-pole low-pass Bessel anti-aliasing filters are applied to all four SCM signals (−3 dB at 60 kHz). SCM-LF signals are then separated into three low gain and three high gain channels, with a 15 × relative gain difference. In total, the DFB presents twenty-six analog signals to the analog to digital converters (ADCs). Table 3 summarizes these signals.

To digitize twenty-six signals at 150 kS/s and maintain a low-power, low-mass, radiationtolerant design, the DFB makes use of the Teledyne SIDECAR application specific integrated circuit (ASIC) (Loose et al. 2005). The SIDECAR was designed to support compact focal plane electronics for space telescopes. A SIDECAR ASIC is currently operating on the Hubble Space Telescope Advanced Camera for Surveys, and the James Webb Space Telescope mission will use make use of the SIDECAR. The DFB does not engage most of the SIDECAR’s focal plane-specific capabilities, instead treating it as a bank of 32 16-bit ADCs (analog to digital converters).

After analog to digital conversion, digital signal processing occurs within a Microsemi RTAX4000 FPGA, converting the twenty-six available data streams into a range of time-domain and spectral-domain data products. Immediately after analog-to-digital conversion, all DC-coupled single-ended and differential signals have their cadences reduced to 18.75 kS/s using an 8-point boxcar average, and average voltage signals (DC- and AC-coupled) in the plane of the heat shield are calculated digitally: *V_Avg_* = (*V*_1_ *+ V*_2_ *+ V*_3_ *+ V*_4_)/4.

All signals then enter one of two cascading digital filter banks (low-speed for 18.75 kS/s data, high-speed for 150 kS/s data). The filter banks apply a fifth-order finite impulse response (FIR) Bessel filter to the incoming data. They then apply an averaging FIR and decimate the data by a factor of two. This process operates recursively, resulting in multiple low-speed waveform data streams at sample rates of 18.75/2*^N^* kS/s for *N* from 0 to 14. High-speed waveform data streams have sample rates of 150/2*^N^* kS/s with *N* from 0 to 6. SCM-LF data streams start in the high-speed digital filter cascade and are passed to the low-speed digital filter cascade upon reaching 18.75 kS/s in order to make the SCM-LF data available to all low-speed and high-speed time- and spectral-domain data products. Further details on the digital filters appear in Cully et al. (2008) and Ergun et al. (2014). The DFB uses the output of these cascading digital filter banks to generate survey waveform data, which has a low cadence (relative to the ADC sampling frequency), but which has continuous coverage over the Solar Probe Plus orbit.

The DFB also produces competitively selected snapshots of high-speed waveform data using the DFB burst memory (DBM). DBM snapshots are *Nx* ~3.5 s long and are comprised of six (selectable) waveform channels captured simultaneously, where *N* is determined by the selected sample rate 150/2*^N^* kS/s. The DBM is capable of producing far more data than the DCB internal memory can store. Therefore, data is down-selected using a competitive buffer scheme. In this scheme, there is one circular ingress buffer constantly being filled, several hold buffers which retain high-quality data, and an egress buffer which is read out to the DCB. Because emptying the egress buffer is a slow process relative to filling the ingress buffer, data in the ingress buffer is periodically assigned a buffer quality. Each time an ingress buffer quality is calculated, it is compared with the hold buffer quality values. If the ingress buffer quality exceeds a hold buffer quality, the ingress buffer is promoted to hold buffer status. The displaced hold buffer data will either be demoted to a lower hold buffer, or else it will be discarded. Whenever the egress buffer is emptied (completely read out to the DCB), the hold buffer data with the highest quality is promoted to egress status. In this way, DBM data quality is competitively assessed, and approximately 1 % of the highest quality data is sent to the DCB for storage and potential telemetry to the ground.

The DBM has six hold buffers, split into two parallel competitive paths. Side A contains hold buffers which compete on the basis of the Coordinated Burst Signal (CBS). Side B contains hold buffers that compete on the basis of DFB-assigned quality. These two sides alternate in their access to the egress buffer. For Side B, buffer quality is assigned by the DFB as follows. One of the six selected DBM channels is designated the ‘trigger’ channel. For every *m* samples of the trigger channel (called a slice), the eight points with the largest absolute value amplitudes are selected. The buffer quality is then assigned using one of three schemes: (1) the maximum of these eight points, (2) the average of these eight points, or (3) the average of the lower seven of these eight points. This third configuration is intended to reduce the number of dust impact voltage spikes captured by the DBM. See Zaslavsky et al. (2012) for a description of dust impact voltage spikes observed by the STEREO spacecraft in the solar wind and Malaspina et al. (2014) for a voltage spikes observed by the Wind spacecraft in the solar wind. As the ingress buffer fills, slice quality flags are calculated continuously. While successive slice quality flag values are increasing, designation of the ‘largest’ quality flag is reserved until slice quality begins to decrease. In this way, the DBM can be re-triggered by later, more interesting data.

The DFB also produces several spectral data products. The first of these is the band-pass filter bank (BP) data. The BP data products on Solar Probe Plus are direct decedents of those produced on THEMIS (Cully et al. 2008) and the Van Allen Probes (Wygant et al. 2013). BP data is produced from band-passed waveform data, generated by taking the difference between two adjacent digital filter bank cascade outputs. The low-speed and high-speed digital filter bank cascades produce 15 and 7 streams of BP waveform data (respectively). For each *Q* samples of bandpass waveform data, the maximum absolute value and the average absolute value are calculated (*Q* is configurable), resulting in 15-bin low-speed BP spectra and 7-bin high-speed BP spectra. The BP spectra have coarse frequency resolution but high time resolution. Up to 4 low-speed and 4 high-speed channels of BP data are selectable, with any low-speed or high-speed (respectively) waveform channel as a source. BP data is used as one DFB input to the coordinated burst signal.

The DFB also produces power spectra and cross spectra using a windowed FFT (Fast Fourier Transform) algorithm, for up to 4 low-speed and 4 high-speed channels. In all cases, transforms are calculated on 1024-point segments of waveform data (9.375 kS/s for low-speed, 150 kS/s for high-speed), with a Hanning window applied. The FFT algorithm produces both real (R) and imaginary (*I*) spectral components. Given input signals 1 and 2, power spectra (*P*) are calculated as $P1_{k}=\left(R1_{k}^{2}+I1_{k}^{2}\right)$ and $P2_{k}=\left(R2_{k}^{2}+I2_{k}^{2}\right)$ for frequency bin k. Real and imaginary cross-spectral components are calculated as *RX_k_* = (*R*1*_k_R*2*_k_ +I*1*_k_I*2*_k_*) and *IX_k_* = (*R*2*_k_I* 1*_k_* − *R*1*_k_ I*2*_k_*). Real and imaginary cross spectral components can be used to derive the coherence and phase between signals 1 and 2.

Power spectra and cross-spectra results are averaged into pseudo-logarithmically spaced frequency bins, with either 56 or 96 frequencies. With 56 frequencies, the bin width (*df/f*) varies between 6 % and 12 %. With 96 frequencies, *df/f* varies between 3 % and 6 % per bin. Narrow band signals can be excluded by applying a spectral mask prior to frequency binning. This capability may be exercised to exclude, from spectral products, narrow-band electromagnetic interference due to the spacecraft or other instruments.

DFB power spectra and cross-spectra can be time-averaged, where the amount of timeaveraging is configurable and may be set independently for low-speed and high-speed products. Time-averaging sets the number of 1024 point spectra to average when reporting a single spectral result. Low-speed spectral products have full data coverage, while high-speed spectral products can sample up to 12.5 % of the incoming data.

The DFB also generates a calibration signal for the SCM. The calibration signal consists of the sum of two sine waves, where the frequency of one sine wave is fixed at 9.6 kHz and the frequency of the second wave changes every 64 wave cycles. This calibration signal is designed to enable in-flight characterization of the gain and phase response of the SCM across the lower range of its sensitive frequency range.

In its normal mode of operation, DFB will produce a stream of ‘Survey’ data over the entire science orbit, that will be transmitted to the Digital Control Board (DCB) and become available for telemetering to the ground. It will also produce a stream of ‘Burst’ events from the DFB burst memory (DBM). A typical Survey Mode for the DFB might be to transmit 2 antenna voltages, 3 electric field measurements (differential voltages), and the 3 SCM magnetic field measurements all as waveforms at 128 S/NYs. This rate might be stepped down to 64 S/NYs or lower at higher altitudes where the physical timescales are longer. In addition, Survey Mode will include power spectral density measurements at the native spectral resolution (*df/f* ~ 3–6 %) and band-pass filter bank (BP) data, as described above. Both AC (75 kHz Nyquist) and DC (~4.7 kHz Nyquist) spectra and BP data will be provided, from some combination of sensors. Table 4 shows a nominal stream of Survey Mode data.

This configuration would give good waveform coverage of electric and magnetic fields and density fluctuations to the convected ion scales over the whole orbit, and spectral density measurements to the electron cyclotron frequency.

In Burst Mode, the DFB will provide fast (up to 150 kS/s) of similar waveform quantities (3E, and 3 *δ*B) and also transmit the cross-spectral matrices described above. These data would be in addition to Survey Mode data.

#### 2.2.3 Time Domain Sampler (TDS)

As described above, in order to increase overall mission reliability, the FIELDS instrument was split into two parts, FIELDS1 and FIELDS2. Each half has some instrumentation, control over some of the FIELDS antennas and a power supply. The core of FIELDS2 is the TDS. As originally planned, the TDS subsystem was a single board processor controlled burst acquisition system designed to collect transient wave phenomena from the FIELDS electric and magnetic sensors. The new design added an interface to the SPP spacecraft command and data handling system, control of one of the two FIELDS DC magnetometers (the in-board MAGi), control of one of the two FIELDS antenna electronics boards (AEB2 controlling antennas V3 and V4) and control of one of the two FIELDS power supplies (LNPS2). In addition, the TDS also maintains a communications interface with the SWEAP instrument. The FIELDS TDS derives heritage from a similar instrument on the STEREO spacecraft (Bougeret et al. 2008).

**Fig. 15:**
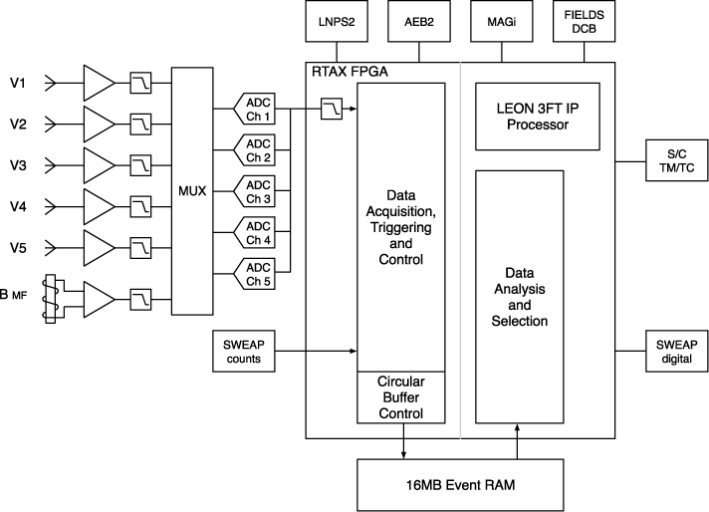
Block diagram of the TDS. The TDS processes 6 input signals at 1.92 MSa/s and produces waveform capture events organized and telemetered by quality. TDS also has a command and data handling (C&DH) interface to the spacecraft computer and can take over in the event of failure on the FIELDS1 side

As the core of the FIELDS2 side of FIELDS, the TDS performs a number of typical data processing functions. Especially crucial is the way the TDS keeps track of time. In normal circumstances, the spacecraft interface provides the TDS with precise information as to the mission elapsed time (MET). The TDS can use this time in its time-stamping of data. However, in order to synchronize the two halves of FIELDS, the TDS also receives information for the FIELDS1 side (from the DCB) indicating the precise MET as it was acquired by the DCB. In normal circumstances, the TDS uses the MET received from DCB as the source of its internal clock. In addition, in order to reduce noise and coordinate measurements, the various clocks throughout the FIELDS suite are synchronized. The FIELDS1 DCB produces a set of master clocks, all derived from a single internal high frequency master clock. These clocks go to the data acquisition samplers and to the various chopping power supplies. In normal circumstances, the TDS operational clocks are taken from the DCB. In the case where the DCB clocks are unavailable, the TDS also includes its own internal clock. The TDS block diagram is shown in Fig. 15.

The Time Domain Sampler instrument (TDS) makes rapid samples of waveforms for the study of high frequency waves. The rapid simultaneous sampling of five channels to nominally include two orthogonal electric dipoles, a single ended electric monopole, the radial (sunward) component of the electric field provided by V5, and a single axis from the search coil magnetometer allows the study of waveforms, their distortions, and, through ground-based spectral analysis, a frequency determination which is far more accurate than an on-board filter analysis system.

The highest sampling rate is about two million samples per second (1.92 MSa/s giving a ~1 MHz Nyquist frequency) with several lower commandable rates (e.g. 480 kSa/s, 120 kSa/s and so on). The sampling clock is derived from the FIELDS master clock provided by the DCB. At the top sampling speed, the TDS has 160 Mbps of total throughput while its nominal share of the FIELDS downlink rate is only of the order of a few hundreds of bps. The TDS achieves this large reduction in bit-rate while maintaining high scientific return by selecting events for transmission to the DCB and the ground intelligently.

Waveform events are usually triggered with a peak value in the center of the event. The duration of time-series events is commandable with a typical length of 65,536 samples or about 33 ms. Once an event is acquired, TDS flight software evaluates the event ‘quality’. Events with the highest quality will be selected for transmission to the DCB and then possibly to the ground. Each of the five channels is filtered and digitized simultaneously. The analog-to-digital converters (ADCs) provide 16-bits of dynamic range and are linear over the range. The noise level is less than 30 μV RMS (at 100 kHz) at the input to the preamplifier. The largest signal obtained is about 1 V RMS. The commercial ADCs are protected from latch-ups by a circuit breaker which shuts off power when high current is detected, allowing parasitic currents within the device to dissipate. The TDS processor will normally turn the converters back on after a programmable cooling-off period (nominally 5 seconds).

The TDS also produces a steady stream of high-value information with very little impact on FIELDS telemetry. Once per minute, TDS will give the peak value observed on each channel (during the preceding minute), the mean value, the RMS power value and zero-crossing counters which give an indication of wave frequency and the number of dust impacts in the period. 100 % of the incoming data stream (at 1.92 MSa/s) will be processed here. Examination of this low bit-rate stream will allow after-the-fact selection of events saved in the massive FIELDS burst memory.

While the TDS is a “wave” instrument, as part of the TDS, one of the data acquisition channels will be devoted to counting particles. The TDS includes an interface to the SPP SWEAP instrument. SWEAP will provide the TDS with a signal line indicating particle counts. Each pulse received will indicate a particle collected by a part of SWEAP and will result in incrementing a counter in the TDS. Each time a TDS waveform voltage is sampled (at 1.92 MSa/s), the incrementing counter will be latched and sampled. In this way, TDS events will provide a snapshot of voltage as a function of time as normal and, in addition, will provide a high resolution picture of the simultaneous particle flux as a function of time. This will give an unprecedented view of wave particle correlation.

In addition to the SWEAP particle counts, FIELDS2 and SWEAP will exchange timing and messages to allow SWEAP data acquisition to be synchronized with the FIELDS master clock. The messages will allow both SWEAP and FIELDS to collect and identify periods of high activity and interest.

#### 2.2.4 Radio Frequency Spectrometer (RFS)

The RFS is a dual channel digital spectrometer, designed for both remote sensing of radio waves and *in situ* measurement of electrostatic fluctuations. The RFS receives inputs from the V1–V4 electric field antennas, using the high frequency output of the FIELDS electric field preamplifiers. Both RFS channels are digitally sampled simultaneously, allowing for calculations of auto spectra for each channel and cross spectra between the two channels. Using multiplexers to select antennas, each RFS channel can use as input either the difference between any two antennas (dipole mode) or the difference between any antenna and spacecraft ground (monopole mode). In addition to the electric field antennas, the single axis MF winding from the search coil may also used as an input to the RFS.

The RFS analog electronics are physically located on an isolated segment of the FIELDS DCB circuit board. RFS digital signal processing (DSP) is handled by the DCB FPGA and the DCB flight software. A functional block diagram of the RFS is shown at the top of Fig. 17 as a subsystem contained within the overall DCB block diagram. In the figure, one RFS channel (Channel 1) is shown, while the identical channel is hidden below.

The sensitivity and dynamic range requirements of the RFS are driven by the expected levels of the input signals. Through careful component selection and board layout, the RFS design has been optimized for low noise performance, with the aim of observing the galactic synchrotron spectrum above the instrumental noise level. Observation of the galaxy will offer an absolute calibration source and enable accurate intercalibration of the RFS with other spacecraft.

In the inner heliosphere, the intensity of solar radio emissions will be greatly enhanced, as the Solar Probe Plus spacecraft will be much closer to the source regions of the radio emission. The expected intensity of the largest Type III radio bursts, the strongest radio signals in the RFS frequency range, determines the highest anticipated signal levels seen by the RFS. Together, the galactic signal and the largest expected Type III radio bursts determine the required dynamic range of the RFS receiver. The large dynamic range is achieved with a 12-bit ADC and the ability of the RFS to operate in both high and low gain modes, for small and large signals respectively. In addition to the remote sensing signals (radio bursts and the galaxy), the RFS makes *in situ* measurements of the quasi-thermal noise spectrum (QTN), generated primarily by ambient electrons. Analysis of the QTN spectrum allows for very accurate determination of the total electron density as well as estimates of electron temperature and other plasma properties (Meyer-Vernet and Perche 1989).

The RFS samples the HF preamp output at the FIELDS master clock frequency *f_s_* = 38.4 MSa/s (note that 38.4 MSa/s = 150 kHz × 256), yielding a Nyquist frequency of 19.2 MHz. This cadence is determined by the EMC control plan for the SPP spacecraft. The EMC plan requires that power converters operate at specific, well-controlled frequencies, using multiples of 50 kHz starting at 150 kHz. This frequency specification limits the power supply-generated noise to specific frequency channels, enabling a “picket fence” scheme (Bougeret et al. 2008) where noise-free measurements can be made between the narrowband power supply frequencies and their many higher order harmonics, as described above.

**Fig. 16:**
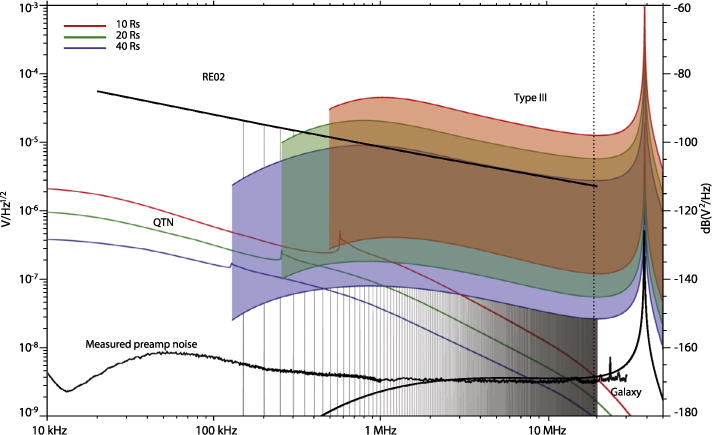
Expected signal levels across the quasi-thermal noise and radio frequency spectrum. Three colored bands show the expected intensity of radio emission associated with interplanetary type III radio bursts and 40 *R_S_* (*lavender*), 20 *R_S_* (*green*), and 10 *R_S_* (*orange*) and spectra of quasi-thermal noise (same color scheme). The spacecraft-level RE02 EMC level is shown as a *solid black line.* Narrow-band ‘spikes’ rising to the RE02 level show the allowable noise contamination (per system EMC specification). The FIELDS Radio Frequency Spectrometer (RFS) instrument will measure this spectrum and reject the noise signals using a Polyphase Filter Bank

**Fig. 17:**
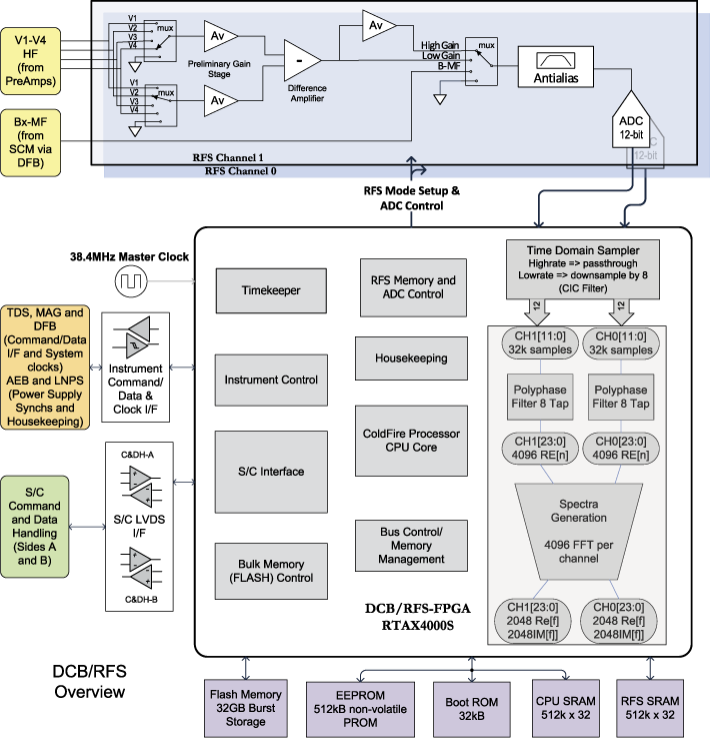
Block diagram of the DCB and RFS, which occupy a single board in the MEP. The DCB is the primary data processing and management module, and interface to the spacecraft C&DH system. RFS uses the DCB computer to perform signal processing of radio frequency measurements

To measure the weakest type III radio bursts, FIELDS needs sensitivity down to the level of the galactic synchrotron spectrum (Novaco and Brown 1978; Manning and Dulk 2001), which is approximately a few $\text{nV}/\sqrt{\text{Hz}}$ at ~ 1 MHz. In general, conducted and radiated noise generated by spacecraft and instrument subsystems exceeds these levels by orders of magnitude. This can be seen in Fig. 16, which shows several estimated signal levels together with the spacecraft electric radiated emission (RE02) requirements. Estimates of interplanetary type III emissions, plasma quasi-thermal noise, and the galactic spectrum all fall 30 or 40 dB below the peak RE02 levels. The required measurement levels are achieved by levying a spacecraft-wide electromagnetic cleanliness (EMC) program that includes maintaining frequency control on all DC-DC power converters.

The DSP signal chain for the RFS starts with a time series of voltage measurements recorded by the ADCs. These time series contain the physical signals of interest as well as the power supply harmonics, which must be removed via spectral processing. Ordinary fast Fourier transform (FFT) techniques are subject to spectral leakage, which would cause the power supply noise to spread from its narrow frequency peaks and overwhelm the signal throughout the RFS frequency range. To reduce this spectral leakage, the RFS implements a polyphase filter bank (PFB) digital signal processing algorithm (Vaidyanathan 1990), followed by a standard FFT. The PFB algorithm weights the data with a windowing function, splits the data into *N* blocks of equal size (“taps”), then adds the taps together to produce a single time series which can then be passed to a standard FFT algorithm. The combined PFB-FFT process optimizes the leakage response of the resulting spectra, effectively isolating the noise in specified frequency bins and preserving the noise-free gaps necessary for making physics measurements.

The nominal length of a RFS sample is 32,768 samples. Using an 8 tap PFB, this results in a 4,096 point time series for the FFT, which in turn yields 2048 positive frequencies. The full resolution spectra would be too large to store and telemeter, and so select frequencies are extracted from the full resolution spectra and stored in memory for downlink. Both autocorrelation and cross correlation measurements are produced from the selected bins of the spectra.

The RFS operational frequency range is 10 kHz–19.2 MHz. This frequency range is subdivided into the LFR (10 kHz~2.4 MHz) and HFR ranges (~1.6 kHz–19.2 MHz), with the primary science of the LFR consisting of the *in situ* QTN measurement, while the HFR focuses on remote sensing. The LFR sampling cadence is reduced from 38.4 MHz to *f_s_* = 4.8 MHz, using a Cascade Integrator Comb (CIC) filter to anti-alias and downsample by 8. Because the frequency resolution of FFT algorithms is equal to *f_s_/N*, the lower *f_s_* allows for better frequency resolution for LFR frequencies while using an identical DSP signal chain. For both the LFR and HFR, the chosen frequencies allow for a relative frequency spacing Δ*f/f* of approximately 4.5 % throughout their respective frequency ranges.

#### 2.2.5 Data Control Board (DCB)

The DCB is the primary controller of the FIELDS instrument. It serves as the primary link between the spacecraft (S/C) and the FIELDS instrument, receiving, decoding and distributing S/C commands to the FIELDS subsystems. Note that as described in Sect. 2.2.3, the TDS subsystem can recover some C&DH capability should the DCB or its power supply fail. The DCB operates autonomously using both Absolute and Relative Time Sequences (ATS and RTS), loaded from the ground prior to each solar encounter. The DCB processes instrument data into two streams, called Survey and Burst, storing the latter in a 32 GB Flash memory. At commanded downlink rates approaching 245 kbps, the DCB selects, compresses and mixes Survey and Burst data into the S/C telemetry stream.

The DCB consists of an embedded processor (CPU) with associated CPU memory (PROM, SRAM, EEPROM) and a dedicated bulk memory array (Flash). The DCB block diagram is shown in Fig. 19. The CPU is a Coldfire 32-bit IP-Core implemented in a radiation- hard RTAX-4000 FPGA. The startup Flight Software (FSW) is stored in a radiation hard 32 kB PROM. Operational software, on-board scripts, tables and other parameters reside in a 512 kB EEPROM, and are transferred to the 2 MB SRAM at start up. Instrument data messages are transferred at 4 Mbps to the SRAM via Direct Memory Access (DMA). The analog housekeeping, instrument control, S/C Interfaces, bulk memory controller subsystems and Radio Frequency Spectrometer logic are also implemented in the same FPGA.

The FIELDS instrument operates synchronously with the DCB managing the clocks and timekeeping. The 38.4 MHz Master Clock, resident on the DCB, provides all FIELDS instrument system clocks as well as the power supply synchronization and interface signal timing. The 38.4 MHz operation, common across the FIELDS subsystems, results in deterministic noise bands. Much of this ‘picket fence’ noise is either removed by selective filtering or recognized by the upstream data processing algorithms.

DCB FSW interfaces with the AEB, RFS, LNPS, DFB, TDS and MAG boards, routing commands from internal ATS and RTS scripts as well as commands from the S/C. FSW collects completed CCSDS packets from the DFB and TDS, then routes these by APID to either the telemetry (Survey) or to the Flash memory (burst). FSW controls the AEB and LNPS, collecting housekeeping analog data, limit monitoring and telemetering them. FSW collects MAG vectors, averages them and generates CCSDS packets at commanded data rates. FSW operates the RFS FPGA logic (as detailed in Sect. 2.2.4), collecting waveforms and performing FFTs. FSW performs median-filtering and production of CCSDS packets with High and Low Frequency spectra, cross spectra, phase and coherence data. FSW also performs Peak Tracking of the Low Frequency data to determine the plasma frequency and thus select the proper high resolution channels to telemeter.

The DCB exchanges burst status, electric and magnetic field information with the S/C and keeps track of Mission Elapsed Time (MET) via S/C messaging. An important part of FIELDS operations is the coordination of bursts by FSW. In order to maximize the collection of the most important and interesting scientific data, the FSW uses a linear combination of incoming data from DFB, TDS, RFS and SWEAP and a table of commandable weighting factors to determine when FIELDS should collect and save burst data in concert. This Coordinated Burst Signal (CBS) is calculated at 4 times/(NY second) and sent to both FIELDS1 and FIELDS2 and the S/C within the second.

The internal solid state recorder allows for the continuous collection of high-data rate acquisitions, the most interesting of which are selected for downlink via ground command. The 32 GB memory is composed of four separate Flash memory modules, each independently switched. FSW is capable of concurrently writing data to, and reading data from, the Flash memory at 2 Mbps. Individual Flash blocks which repeatedly fail are marked ‘off’ the FSW and skipped in subsequent processing. FSW references the Flash memory via a Virtual-to-Physical memory map, allowing large bad sections to be mapped out of the address space. A hardware-based scrubbing subsystem automatically corrects single-bit errors and flags multi-bit upsets. FSW tracks bad-blocks and provides Flash Read and Write pointers in telemetry.

DCB components have been selected to survive the seven year minimum mission length in the harsh solar environment which imposes significant radiation exposure and thermal stresses. High-quality parts (radiation hard FPGA and single-event upset (SEU) immune CPU memories) minimize the probability of upset during the crucial observation periods and maximize the probability of successful operation over the course of the mission.

### 2.3 Low Noise Power Supply (LNPS)

A central part of the FIELDS instrument is its low noise DC-to-DC power supply (LNPS). In order to allow FIELDS to make sensitive measurements of nature, the power supply must not get in the way. The first line of defense is that the chopping power supply must be synchronized such that all lines of noise that are produced are controlled to lie on the 150 kHz picket fence. In addition to that, the supply has been laid out to provide voltages that are stable and isolated such that the various loads have independent grounds or returns.

As part of an effort to increase the overall reliability of the FIELDS instrument, the original concept of a single FIELDS power supply was split such that half of FIELDS is powered by one power supply (LNPS1) while the other half is powered from a second independent supply (LNPS2). Each of the two supplies has a separate independently switched spacecraft power supply. In addition, each of the supplies passes heater power to various remote electronics modules.

LNPS1 provides power for the Radio Frequency Spectrometer (RFS), the Digital Fields Board (DFB), the out-board DC Magnetometer (MAGo), the Search Coil Magnetometer (SCM) assembly, an Antenna Electronics Board (AEB1) and the central Data Control Board (DCB). Supplies produced are at 1.9 V, 3.3 V, 4 V, 5 V, +/−6 V, + /−12 V and +/−100 V. The total delivered secondary power ranges from 7 watts quiescent to about 11 watts at peak. Heater power is provided directly to MAGo and the SCM. Somewhat smaller, LNPS2 provides power for the Time Domain Sampler (TDS), in-board DC Magnetometer (MAGi) and a second Antenna Electronics Board (AEB2). Supplies produced are at 1.5 V, 3.3 V, 5 V, +/−6 V, +/−12 V and +/−100 V. The total delivered secondary power ranges from 4 watts quiescent to about 7 watts at peak. Heater power is provided directly to MAGi.

In both supplies, the first stage is a pre-regulator taking unregulated voltage from the spacecraft bus to make a very stable 12 V supply using a synchronous switching controller driving a pair of FETs that charge a buck-coil circuit. The well regulated 12 V supply is then used to power three separate pulse width modulator (PWM) circuits that provide square waves to drive one pair of FETs each which in turn drive two transformers each, for a total of six transformers in all. The second stage PWMs are configured to run flat out—making 50/50 square waves. The pre-regulator and the first of three PWMs are synchronized to chop at 150 kHz using a 600 kHz square wave derived from the master clock within the DCB. The second and third PWMs are then slaved to the first PWM such that all run at 150 kHz. If the incoming synchronization clock should fail, all four chopping circuits will run independently at approximately 135 kHz. Soft starts of the three PWM’ will cause them to turn on with slight delays, minimizing in-rush current as well as providing a desired delay in the turn on of the +/−100 V supply used by the AEBs.

Most of the supply return lines are independent from one another such that the various instruments will be able to ground them locally in order to minimize the effects of noise. Both LNPS boards include in-rush filtering and LCs on all outputs. Common mode chokes (CMC) are installed in series with all instrument supplies such that, even in the cases where two instruments share a supply, they will be isolated with CMCs.

## 3 FIELDS Science Operations

### 3.1 Concept of Operations

On-orbit, the FIELDS instrument is relatively simple to operate. When power is applied, FIELDS will configure itself into a default survey mode. Using a commanded non-volatile Absolute Time Sequence (ATS) covering an entire perihelion pass, instrument software will use Spacecraft Time and determine the preferred instrument configuration for any point in the orbit. Much of the instrument commanding is facilitated by up to 64 ground-loaded nonvolatile Relative Time Sequences (RTS) that accomplish different instrument configurations. For example, an RTS might set the MAG1 and SCM sampling to the same rate for the purpose of cross-calibration, hold for a period and return the rates to nominal values.

**Fig. 18:**
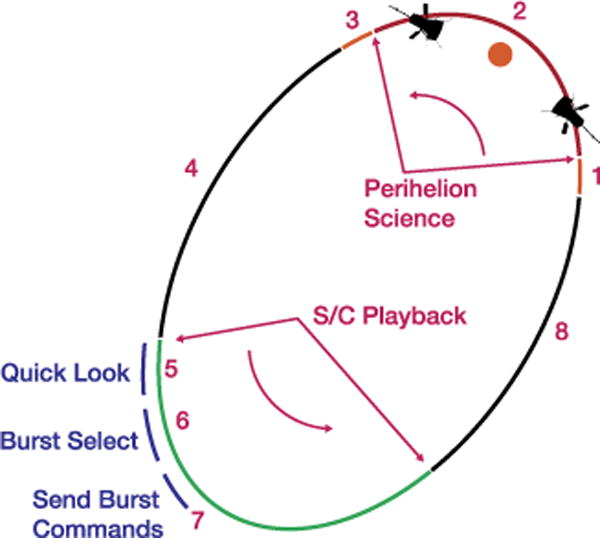
FIELDS orbit sequence showing data acquisition and playback intervals

As shown in Fig. 18, FIELDS operational concept contains a number of steps during the nearly 90 day orbit.

As SPP descends toward perihelion (e.g. ~8 days prior), FIELDS is set into Calibration mode, with a low 2 kbps output rate to the spacecraft. After Calibration, Antenna bias sweeps will measure properties of the sensors (pre-encounter). Ground operators verify that the ATS is loaded and operating. A z-axis slew is required for FIELDS calibration once per orbit at solar distance >0.25 AU and <0.82 AU. It involves 2 complete 360 degree rotations about the s/c z-axis at the max rotation rate available with the s/c wheels. The rotations can be executed in + /*−* direction for wheel momentum balance.
At about 6 days to perihelion, FIELDS will enter High Rate Science mode, in which it will send about 20 kbps of prioritized Survey data to the spacecraft SSR in real-time, while it stores about 100 kbps of High Rate Science data to the internal 32 GB solid state recorder.
After the encounter, FIELDS is commanded into Calibration mode followed by postencounter Antenna bias sweeps. FIELDS will return to low rate Survey mode and can be turned off if needed.
While off, FIELDS sensors will be kept warm using spacecraft-provided heater power. As downlink opportunities approach, Spacecraft will transmit prioritized Survey data from the S/C SSR to the ground.
FIELDS SOC processes and performs preliminary analyses on the Prioritized Survey data, including Quick Look plots, and distributing to the team.
6. At aphelion, the FIELDS team will convene to examine the playback data and determine periods of special interest from the preceding perihelion. Command sequences will be generated to select this special data for playback as SPP approaches the next perihelion. In addition, the team may prepare modes of operation for the next encounter; and identify configuration or software changes needed.
Following event selection, commands are sent to playback sections of the FIELDS internal solid state recorder to the Spacecraft SSR, and the Spacecraft will forward these data to the ground, in step 8. Commands sequences for the next Perihelion pass are also sent to the MOC for uplink.
FIELDS high rate data is available for transmission from the Spacecraft SSR to the ground. During this period FIELDS will continue to operate in low-rate survey mode, as permitted by downlink opportunities.

### 3.2 Science Operations Center Concept

FIELDS operations will be managed through the FIELDS Science Operations Center (SOC), which will be located at UC Berkeley. The FIELDS SOC has two roles: providing the command, telemetry, and ground support (CTG) for the FIELDS suite, and serving as the science data center (SDC), making FIELDS data available for the FIELDS team and the community.

The FIELDS SOC CTG module supports instrument command and telemetry handling for both integration and test (I&T) and on-orbit configurations. Commands will be generated by the FIELDS operational team and communicated to the instruments via emulators, using a GSEOS (Ground Support Equipment Operating System) interface. Communication with the FIELDS MOC will also utilize GSEOS. On-orbit, commands to the FIELDS instruments will pass through the MOC. However, the GSE interface will be developed to be as similar as possible to the final on-orbit interface, so that from the point of view of the SOC generation of commands and production of data, the process will be nearly identical. This will enable a smooth transition from FIELDS I&T, through mission I&T, and on through on-orbit operations.

The commands sent to the FIELDS instrument will vary from orbit to orbit, due to changes in the ephemeris, the selection of burst intervals, and varying availability of data downlink periods. For each orbit, a specific sequence of commands will be generated and sent to the spacecraft, via the CTG. In addition, the CTG will be used to deliver and necessary software or configuration table updates.

**Fig. 19:**
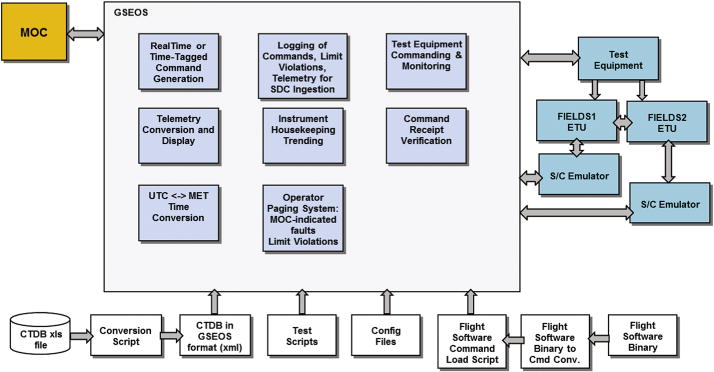
FIELDS CTG data flow diagram

As discussed previously, both the DCB (FIELDS1) and the TDS (FIELDS2) boards have the capability to interface to the spacecraft and send FIELDS data, thereby removing a single point failure of SPP objectives. As shown in Fig. 19, this implies that the FIELDS SOC must similarly develop the capacity to independently command both of the boards.

The FIELDS SOC SDC produces and distributes data products. The production of FIELDS data will be described in the following section.

### 3.3 Data Products and Software

**Fig. 20:**
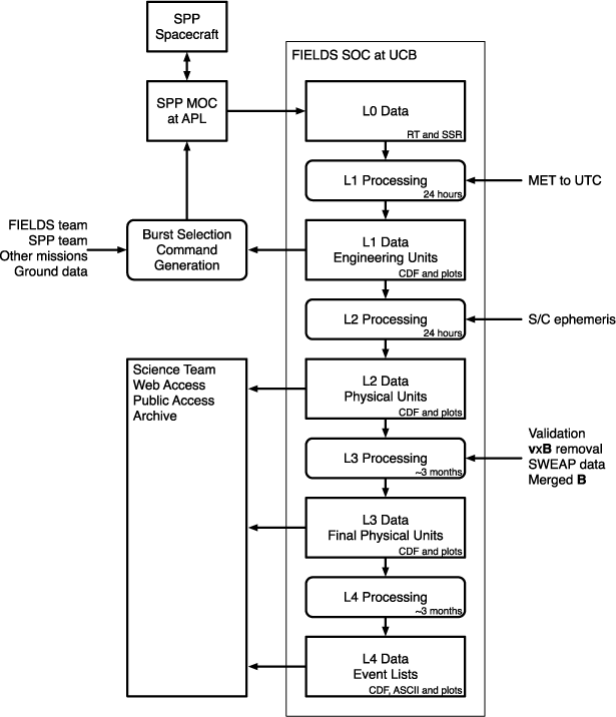
FIELDS data processing flow diagram

Figure 20 shows the basic data processing flow for FIELDS data through the SDC. Starting with the Level 0 raw telemetry data obtained from the SPP Mission Operations Center (MOC), the SOC produces Level 1 data, which consists of time-tagged data products in engineering units (volts, dB), in the spacecraft coordinate system. Data at this level is produced in a fully automated fashion, and so processing can be quick, on the order of 24 hours. Level 1 data is used to make the earliest quicklook plots, and is suitable for selection of burst data intervals.

From the Level 1 data combined with ground based calibration tables, Level 2 data will be produced. Calibrated Level 2 data is presented in physical units $\left(\text{volts}/\text{meter},\text{nT},\left(\text{volts}/m\right)/\sqrt{\text{Hz}},\text{nT}/\sqrt{\text{Hz}}\right)$, and in various spacecraft and heliospheric coordinate systems. As with the Level 1 data, the Level 2 data production process will be fully automated and therefore capable of producing data quickly.

Level 3 data production involves data from other instruments and validation of data, such as incorporating SWEAP velocity data in order to remove the **V** × **B** electric field from the data. At this point, the data from the flux gate magnetometers and the search coil will also be merged to make a single data product. The data will also be inspected and validated by the FIELDS science team, and the validated Level 3 data is suitable to make science plots for publication. Because of this necessary human validation, the Level 3 data process is not fully automated and so will take more time, about 3 months, or roughly one orbit, before being released.

Level 4 data consists of items such as event lists, for phenomena such as interplanetary shocks or radio bursts. Level 4 data will be produced by inspection of the data, and will also be produced and released to the science community on about 3 month, or one orbit, time scales.

All FIELDS science data products at Level 2 and above will be made available in ISTP-compliant CDF files, using the FIELDS webpage hosted at UC Berkeley. The CDF files will also be distributed to public archives such as SPDF/CDAWeb. Quicklook plots of summary science data will be made available via the FIELDS webpage. Software tools for analysis of FIELDS data will be made publically available. These tools will be based on the THEMIS Data Analysis Software (TDAS), which consists of a set of IDL routines which can be used to download, analyze, and make publication quality plots from CDF files. In addition to the THEMIS mission, TDAS software has been recently used to provide access to RBSP/EFW and MMS data. The similarity of the FIELDS data products to those produced by THEMIS and EFI will make development of the FIELDS TDAS routines straightforward.

Users of other software (MATLAB, Python, etc.) will be also able to import and analyze the provided CDF files, although no development of FIELDS-specific routines is envisioned for tools other than IDL and TDAS.

While the exact data products have not yet been finalized, it is likely that CDF files will be produced on day boundaries, with separate files for Survey Mode magnetic field, electric field and antenna voltages, power spectral density and BP data, DFB Burst Mode, RFS, and TDS data.

## 4 Summary

The SPP/FIELDS instrument will make the very first *in situ* measurements of the coronal magnetic field, Alfvén waves and their Poynting flux, MHD turbulence within the Alfvén surface, plasma density and electron temperature, and interplanetary shocks and reconnection in the inner heliosphere. Solar radio emission associated with microflares may be observable for the first time and FIELDS will make measurements that can infer interplanetary dust physics as well.

The era of Solar Probe Plus data promises to be revolutionary for the physics of coronal heating and solar wind acceleration. The SPP/FIELDS measurements will be a central set of observations during this exciting time.

## Figures and Tables

**Table 1:** Level 1 requirements for the FIELDS instrument suite

Measurement	Dynamic range	Cadence	Bandwidth
Magnetic Field	140 dB	100k vectors/s	DC–50 kHz
Electric Field	140 dB	2M vectors/s	DC–1 MHz
Plasma Waves	140 dB	1 spectrum/s	5 Hz–1 MHz
QTN/Radio	100 dB for QTN	1 spectrum/4 s for QTN	10–2500 kHz for QTN
	80 dB for radio	1 spectrum/16 s for radio	1–16 MHz for radio

**Table 2:** Expected typical values for plasma parameters and other derived quantities to be measured by FIELDS at different heliocentric distances. *δB_A_* and *δE_A_* are expectations for large Alfvénic fluctuations. *δE_L_* is the expected level of wave electric field fluctuations given a scaling that assumes constant energy density

Parameters		10 *R_s_*	55 *R_s_*	1 AU
Magnetic Field	*|B*_0_*|~ δ B_A_*	2000 nT	70 nT	6 nT
Electric Field	*|E_c_|* ≤ *v_sw_ δ B_A_*	100 mV/m	30 mV/m	3 mV/m
Density	*n_e_* ~ *δn*	7000 cm^−3^	120 cm^−3^	7 cm^−3^
Electron Temperature	*T_e_*	85 eV	25 eV	8 eV
Solar Wind Speed	*v_sw_*	210 km/s	400 km/s	450 km/s
Alfvén Speed	*v_A_*	500 km/s	125 km/s	45 km/s
Plasma Frequency	*f_pe_*	750 kHz	100 kHz	24 kHz
Electron Gyrofrequency	*f_ce_*	60 kHz	2 kHz	160 Hz
Proton Gyrofrequency	*f_cp_*	32 Hz	1 Hz	0.1 Hz
Convected Debye Length	*v_sw_/*λ*_D_*	4 μs	8 μs	22 μs
Convected Electron Inertial Length	*v_sw_*/(*c/ω_pe_*)	0.3 ms	1.2 ms	5.5 ms
Convected Proton Inertial Length	*v_sw_/*(*c/ω_pi_*)	13 ms	50 ms	250 ms
Convected Proton Gyroscale	*v_sw_*/*ρ_p_*	3 ms	30 ms	200 ms
DC/LF Electric Fluctuations	*δE_A_* ~ *v_A_ δB_A_*	1 V/m	10 mV/m	1 mV/m
Kinetic Electric Fluctuations	*δE_L_*	1 V/m	70 mV/m	10 mV/m

**Table 3:** DFB output signals available to digital signal processing

DFB output signal	Description
*V*_1_, *V*_2_, *V*_3_, *V*_4_, *V*_5_ (DC)	DC-coupled antenna voltages
*V*_1_, *V*_2_, *V*_3_, *V*_4_, *V*_5_ (AC)	AC-coupled voltages
*E*_12_, *E*_34_, *E_z_* (DC, low gain)	DC coupled differential voltages, low gain
*E*_12_, *E*_34_, *E_z_* (DC, high gain)	DC coupled differential voltages, high gain
*E*_12_, *E*_34_, *E_z_* (AC)	AC coupled differential voltages
*B_x_*, *By*, *B_z_* (LF, low gain)	Orthogonal SCM axes, low frequency windings, low gain
*B_x_*, *By*, *B_z_* (LF, high gain)	Orthogonal SCM axes, low frequency windings, high gain
*B_x_* (MF)	SCM × axis, medium frequency winding

**Table 4:** An example of DFB data products in Survey Mode near SPP perihelion. Lower cadences will likely be used when SPP is further from the Sun

Data	Sensors	Cadence
Waveforms	2V,3E,3 *δB*	128 S/NYsec
Power spectra	4 AC, 4 DC	1 spectrum/(4 s)
Band-pass filters	4 AC, 4 DC	1 S/s
